# Clinical Implications and Gender Differences of KCNQ1 p.Gly168Arg Pathogenic Variant in Long QT Syndrome

**DOI:** 10.3390/jcm9123846

**Published:** 2020-11-26

**Authors:** Rebeca Lorca, Alejandro Junco-Vicente, Maria Martin-Fernandez, Isaac Pascual, Andrea Aparicio, Noemi Barja, Elias Cuesta-LLavona, Luis Roces, Pablo Avanzas, Cesar Moris, Eliecer Coto, José Julían Rodríguez Reguero, Juan Gómez

**Affiliations:** 1 Área del Corazón y Departamento de Genética Molecular, Hospital Universitario Central Asturias, Unidad de Referencia de Cardiopatías Familiares-HUCA, 33011 Oviedo, Spain; lorcarebeca@hotmail.com (R.L.); eliascllavona@gmail.com (E.C.-L.); cesarmoris@gmail.com (C.M.); eliecer.coto@sespa.es (E.C.); josejucasa@yahoo.es (J.J.R.R.); juan.gomezde@sespa.es (J.G.); 2 Heart Area, Hospital Universitario Central de Asturias, 33011 Oviedo, Spain; ajuncovicente@gmail.com (A.J.-V.); mmartinf7@hotmail.com (M.M.-F.); apariciogavilanes@gmail.com (A.A.); noeminbg@gmail.com (N.B.); avanzas@secardiologia.es (P.A.); 3 Instituto de Investigación Sanitaria del Principado de Asturias, ISPA, 33011 Oviedo, Spain; 4 Anestesiología, Reanimación y terapéutica del dolor, Completo Asistencial Universitario de Salamanca, 37007 Salamanca, Spain; luisrocessoto@gmail.com

**Keywords:** long QT syndrome, inheritable arrhythmogenic disorder, genetic testing

## Abstract

Background: Long QT syndrome (LQTS) is an inheritable arrhythmogenic disorder associated with life-threatening arrhythmic events (LAEs). In general, patients with LQTS2 (*KCNH2*) and LQTS3 (*SCN5A*) are considered to be a greater risk of LAEs than LQTS1 (*KCNQ1*) patients. Gender differences are also important. Series analyzing families with the same pathogenic variants may help in the progress of elaborating strong specific genotype-phenotype management strategies. In this manuscript, we describe the phenotype of seven unrelated families, carriers of the *KCNQ1* G168R pathogenic variant. Methods: we identified all consecutive index cases referred for genetic testing with LQTS diagnosis carriers of *KCNQ1* G168R variant. Genetic and clinical screening for all available relatives was performed. Results: we evaluated seven unrelated families, with a total 34 *KCNQ1* G168R carriers (two obligated carriers died without available EKGs to evaluate the phenotype). All index cases but one were women and three of them presented with aborted sudden cardiac death (SCD) or syncope. The presence of sudden death in these families is notable, with a total of nine unexplained sudden deaths and four aborted SCD. Phenotype penetrance was 100% in women and 37.5% in men. Conclusions: *KCNQ1* G168R is a pathogenic variant, with a high penetrance among women and mild penetrance among men. Risk for LAEs in this variant seems not negligible, especially among woman, and risk stratification should always be carefully evaluated.

## 1. Introduction

Long QT syndrome (LQTS) is an inheritable arrhythmogenic disorder, characterized by a prolonged ventricular repolarization (QTc interval) associated to a higher susceptibility to life-threatening arrhythmic events (LAEs) [1]. To date, more than 17 genes have been described associated with LQTS. However, most pathogenic variants are still identified in the three first described genes: *KCNQ1*, *KCNH2*, and *SCN5A* [2,3], causing LQTS1, LQTS2, and LQTS3, respectively.

Risk stratification in patients with LQTS is crucial. Despite QT measurement limitations, QTc duration is considered a strong predictor of LAEs in numerous studies [4,5]. The arrhythmic risk of patients with concealed LQTS has been shown to be remarkably lower than in patients with overt LQTS [5]. Current guidelines for clinical practice recommend estimating arrhythmic risk in patients with LQTS based not only on QTc duration [6,7,8,9], but also based on specific genotype. Moreover, the clinical manifestations of LQTS may vary according to the different genetic background. Although the affected gene may be a major factor in phenotype, the specific disease-causing variant could also contribute to the clinical severity [10]. In general, patients with LQTS2 and LQTS3 are considered to be a greater risk of LAEs than LQTS1 patients [6,7,8]. However, LQTS1 caused by the pathogenic variant *KCNQ1* A341V presents with an unusual clinical severity [11,12].

The *KCNQ1* gene encodes the α-subunit of the K+ channel Kv7.1, generating the repolarizing IKs current. When heart rate increases, IKs currently plays a crucial role in ensuring proper adaptation of the length of the QT interval. Thus, when IKs is defective, the QT interval fails to shorten appropriately during tachycardia [10]. Therefore, LQTS1 patients are at higher risk of LAEs during sympathetic activation, such as during exercise (especially swimming) and emotions [13]. Heterozygous *KCNQ1* pathogenic variants cause the dominant Romano–Ward LQTS1 and account for the majority of disease-causing variants [2,3,10].

To date, despite these important general genotype risk association considerations, the dream of elaborating strong specific genotype-phenotype management strategies according to the family variant still remains out of reach. Many papers have studied genotype-phenotype relations based on cohorts of index cases carriers of pathogenic variants in the same gene. However, pathogenic variants in the same gen may differ from each other and clinical expression in other relatives may vary from the index cases. Therefore, long series describing several families with the same pathogenic variant may help in the progress of elaborating strong specific genotype-phenotype correlations.

On the other hand, gender differences in LQTS are of utmost importance. Despite the autosomal dominant inheritance of the disease, more than 60% of patients referred for LQTS clinical genetic testing in large series of unrelated LQTS patients are women [2]. Additionally, the risk of LAEs also differs between genders depending on their genotype. For instance, the risk was found to be higher among LQT2 females than LQT2 males, and higher among LQT3 males than LQT3 females [9].

In this scenario, in the daily practice of our reference center for hereditary cardiac pathologies, the clinical data of certain families with LQTS1, carriers of the same pathogenic variant, caught our attention. Therefore, we aimed to describe the clinical presentation of seven unrelated families, carriers of *KCNQ1* G168R, analyzing the penetrance, phenotype, and differences between genders.

## 2. Methods

### 2.1. Study Population

We identified all consecutive index cases referred for genetic testing with LQTS1 diagnosis. Index patients carriers of *KCNQ1* G168R variant were included in this study. All available relatives underwent genetic and clinical screening. 

Clinical data were filed in a custom-made registry and included demographic information, personal and family history of symptoms, arrhythmic events, electrocardiographic parameters, and therapies. 

For the measurement of electrocardiographic parameters, we adopted the methodology introduced by the Long QT Syndrome International Registry [5]. Accordingly, we obtained the first available 12-lead electrocardiogram (paper speed 25 mm/s and voltage settings 10 mm/mV) before therapy when accessible, at stable heart rates close to 60 beats/min during daylight hours to limit the confounding effect of diurnal variability of the QT interval [14]. The QT interval duration was measured in lead DII or V5 and corrected for the heart rate using the Bazett’s formula [15]. 

LQTS penetrance was calculated as the percentage of *KCNQ1* G168R carriers with a positive phenotype, with a prolonged QTc interval (QTc longer than 440 milliseconds for men and QTc longer than 460 milliseconds for women). Two obligate carriers, who died without clinical evaluation or available EKGs to review, as their clinical status was unknown, were excluded for penetrance calculation. Carriers of *KCNQ1* p.Gly168Arg variant (positive genotype) could be divided into three categories: (a) carrier with positive phenotype (prolonged QTc) and with cardiac related symptoms (arrhythmia, syncope, aborted SD, or SD); (b) carrier with positive phenotype (prolonged QTc) and without cardiac related symptoms; and (c) carrier with negative phenotype (normal QTc) and without cardiac related symptoms. 

All patients signed written consent to grant access to their genetic data for investigational purposes and the research protocol followed our institutional ethics guidelines. The study was evaluated by the local Ethical Committee (CEImPA 2020.453).

### 2.2. Genetic Testing

Genetic screening was carried out with DNA samples from the LQTS recruited patients. Gene panel including LQTS-associated genes were NGS sequenced by Ion Torrent semiconductor chip technology in a Ion GeneStudio S5 Sequencer (Thermo Fisher Scientific, Waltham, MA, USA), according to previously described protocols [16,17]. Overall coverage of the gene panel was >95%. Variant Caller v5 software was used to variant identification (Thermo Fisher Scientific, Waltham, MA, USA). Ion Reporter (Thermo Fisher Scientific, Waltham, MA, USA), and HD Genome One (DREAMgenics S.L., Asturias, Spain) software were used for variant annotation, including population, functional, disease-related and in silico predictive algorithms databases. Sanger sequencing was performed for family screening for *KCNQ1* p.Gly168Arg variant in an ABI3130XL sequencer (Thermo Fisher Scientific, Waltham, MA, USA). 

Interpretation of all gene variants with an allele frequency <0.01 was based on the American College of Medical Genetics and Genomics (ACMG-AMP) 2015 Standards and Guidelines [18]. 

### 2.3. Statistical Analysis

Statistical analyses were performed with SPSS v.19 (SPSS Inc., Chicago, IL, USA). Descriptive data for continuous variables are presented as mean + SD and as frequencies or percentages for categorical variables. The chi-square test or Fisher exact test were used to compare frequencies an ANOVA was also evaluates. *p* < 0.05 was considered to be significant.

## 3. Results

From the 109 consecutive index patients (57% were women) referred for genetic testing with SQTL suspicion until 2020, we identified 7 unrelated patients with LQTS carriers of the pathogenic variant *KCNQ1* G168R. 

**Figure 1:**
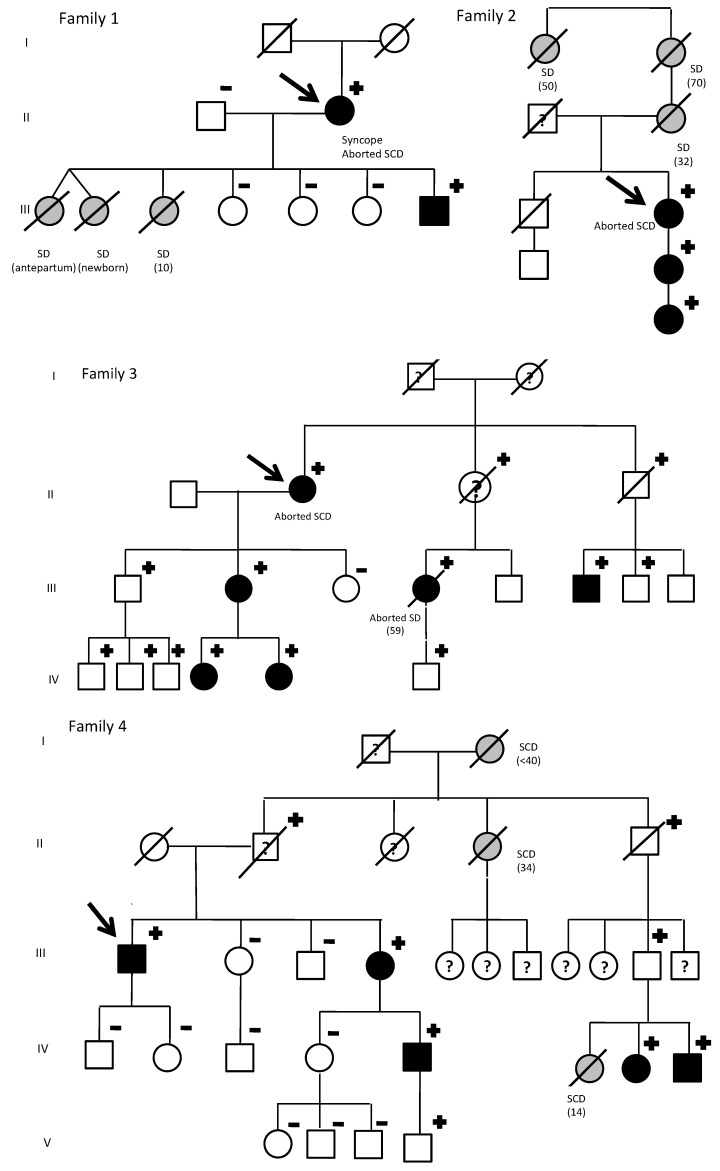

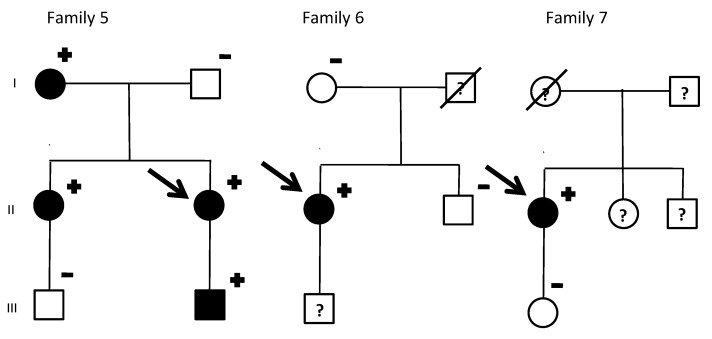
*KCNQ1* G168R carriers: Family pedigrees. SD, sudden death. Age of deceased in patients due to SD, in brackets. Symbols denote sex and disease status: +, carriers; −, noncarriers; without sign, genetic status unknown; box, male; circle, female; black darkened, long QT syndrome phenotype (prolonged QTc in electrocardiogram); grey darkened, unexplained SD; symbol clear, negative phenotype (normal QTc); ?, unknown phenotype; slashed, deceased; arrow, proband.

A total of 34 *KCNQ1* G168R carriers from 7 families were identified. By family screening, besides seven index cases, we identified 27 additional relatives. Two obligate carriers died with unknown phenotype. The 16 non-carriers relatives were discharged from cardiac follow-up. Family’s pedigrees are shown in Figure 1. Main clinical features of index patients are summarized in Table 1.

In family 1, there were three sudden cardiac deaths: two twin girls (one died before birth and the other a few days afterwards) and a girl who died suddenly near the water’s edge on the beach. The index case (a women) was diagnosed due to syncope in 2011 and suffered an aborted SD (polymorphic ventricular tachycardia). Her three other daughters were non-carriers and her son was an asymptomatic carrier, with positive phenotype.

The index case from family 2 presented with an aborted sudden cardiac death due to Torsade de pointes (TdP). Her mother, grandmother and grandaunt had died suddenly the ages of 32, 70, and 50, respectively. She has a daughter and granddaughter, asymptomatic, with positive phenotype. 

The index case from family 3 also presented with aborted sudden cardiac death due to TdP. With family screening, one non-carrier daughter could be discharged. Her other daughter and two granddaughters were asymptomatic carriers, with positive phenotypes. On the other hand, all three grandsons and son carriers had a negative phenotype. Moreover, her niece also suffered an aborted sudden cardiac death. The son of this niece was an asymptomatic carrier and her mother (obligated carrier) had died due to a non-cardiological problem and without clinical evaluation available. Two additional nephews, carriers with negative phenotype, were identified. Their father, obligated carrier had died of a neurological disorder with a normal phenotype. Genetic status of two other nephews was not possible to investigate. 

**Figure 2:**
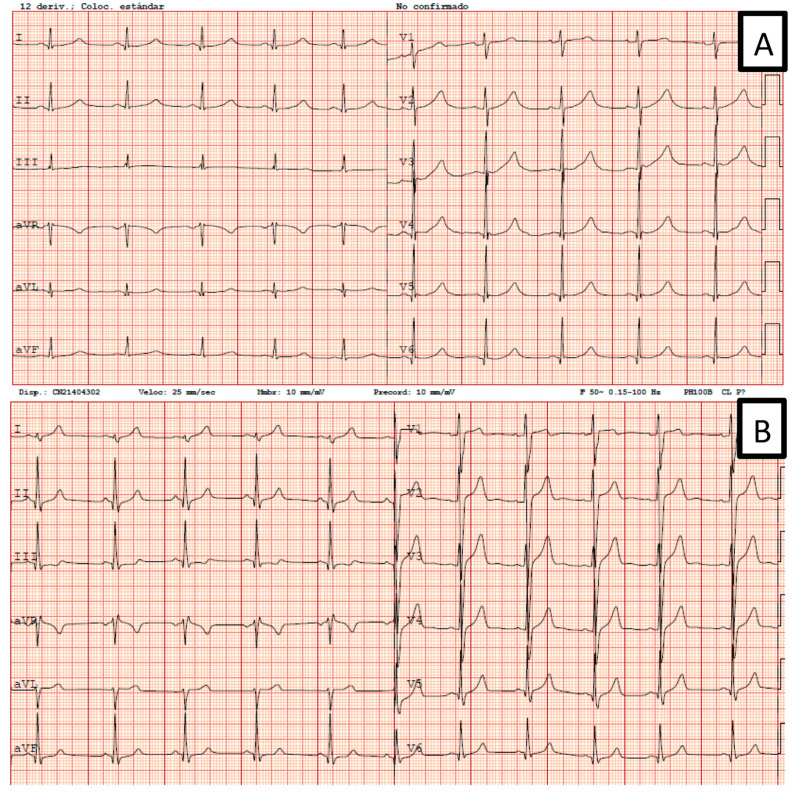
Two EKGs from Family 4. (**A**) Patient IV.5: genotype and phenotype positives. (**B**) Patient V.4: positive genotype and negative phenotype.

In family 4, an abnormal EKG identified the only index male form our cohort. However, remarkably family history of SD was present: her grandmother before 40 years old, an aunt at the age of 34, and the daughter of a cousin, who died at a water park at the age of 14. Family screening identified six additional living relatives that are carriers. His sister was both genotype and positive. She has a son with both genotype and positive and a grandson with positive genotype and negative phenotype (Figure 2). 

His uncle (obligated carrier) had died due to cancer with a normal phenotype. His father, who was also an obligated carried, committed suicide without available clinical evaluation. 

Families 5–7 identified index cases due to QTc prolongation in ECG, without significant family history. Family 5 was also identified by an incidental finding in an ECG from the index case, being all carriers asymptomatic. 

The presence of SCD in these families is notable, with a total of nine unexplained sudden deaths and four aborted SD. From the 34 carriers, 32 were considered for penetrance evaluation (one man and one women who obligated carriers died without unknown phenotype), being 50% men. LQTS1 penetrance was 100% in women, whereas in men the QTc penetrance was estimated as 37.5% (*p* < 0.05). ANOVA analysis (for gender, warning symptoms or family history of SD) was not statistically significant. 

## 4. Discussion

Guidelines for clinical practice recommend estimating arrhythmic risk in patients with LQTS based on QTc duration and genotype [6,7,8]. Genotype-phenotype correlations are still a struggle in most heritable disease. Despite that, an effort should be made in this regard to implement personal medicine in the clinical practice. LQTS can be considered one of the first diseases for which a gene-specific management became possible. For instance, patients with LQTS1 have long been advised to avoid competitive sports and specially swimming [13]. Therefore, it is not striking that two sudden cardiac deaths of our cohort took place while swimming. 

In general, patients with LQTS2 and LQTS3 are considered to be a greater risk of events than LQTS1 patients [6,7,8]. However, not all pathogenic variants on the same gene produce similar clinical phenotypes. For instance, the pathogenic variant *KCNQ1* A341V is associated with clinical severity [11,12], being 80% of patients symptomatic and with over 30% of them experiencing cardiac arrest or sudden death. 

In short, α-subunit can be divided in four parts: the N-terminus, six membrane-spanning domain segments (S1, S2, S3, S4, S5, and S6), two cytoplasmic loops (S2–S3 and S4–S5) and the C-terminus portion. Moss et al. showed that in LQT1 patients both the transmembrane location of the variant and their dominant-negative effect were independent risk factors for cardiac events [19]. In these sense, Shimizu et al. found that patients with transmembrane pathogenic variants had longer QTc and more LAEs than those in the C-terminus [20]. Moreover, Barsheshet et al. found that patients with missense variants located in the transmembrane C-loop (but not in the membrane spanning domain) exhibit the highest risk for LAEs [21]. 

However, to date, neither the localization of the pathogenic variant nor its cellular electrophysiological effects seem sufficient to predict the impact on clinical manifestations [13]. For instance, the “aggressive” *KCNQ1* A341V had a priori low risk characteristic. It is a mildly dominant mutation producing a relatively modest loss of IKs and it is located in the *a priori* “lower risk” membrane-spanning domain (S5–S6). Additionally, in contrast to the above-mentioned studies, another study showed no significant differences between LQTS1 pathogenic variants [22]. 

In this sense, the *KCNQ1* G168R variant analyzed in this study is also located in a membrane-spanning domain (S2). In our cohort a total of 34 carriers of *KCNQ1* G168R were identified. Between these patients with known genotype, there were four aborted cardiac deaths. Moreover, there was a strong family history of sudden death (SD) in four of the seven families, with nine additional SD in women. These findings make us reconsider the benign nature of this pathogenic variant located in the membrane-spanning domain of *KCNQ1* gene. 

On the other hand, although several potential mechanisms trying to explain gender differences in phenotype expression have been reported, many questions remain unsolved. Epigenomic and genomic alterations in the imprinting cluster on chromosome 11p15.5 might affect the regulation and transcription activity of *KCNQ1* gene. Imprinted domain 2 is controlled by imprinting control center 2 (IC2, located in *KCNQ1* intron 10) and regulates the expression of several imprinted genes in *cis* [23]. IC2 is methylated on the maternal allele and thus allows the expression of *KCNQ1* on the maternal allele, while it is unmethylated on the paternal allele resulting in silencing of *KCNQ1* [24,25]. However, loss of methylation of the IC2 can lead to different expression of *KCNQ1*. It has been described that by affecting *KCNQ1* transcription, genetic variants may result in *KCNQ1* haploinsufficiency and LQTS [23]. 

As advanced in the introduction, more than 60% of index patients referred for LQTS clinical genetic testing in large series of unrelated LQTS patients were women [2]. However, these findings seem difficult to explain for a disease with an autosomal dominant inheritance and even more so considering that young men present a more aggressive phenotype and should be referred to cardiology earlier. In addition, a significant deviation from expected Mendelian ratios of allele inheritance (transmission ratio distortion, TRD) in genotyped LQTS families, with an excess of variant carriers and female predominance, has been reported [26]. In this sense, Itoh et at. investigated the parental transmission in LQTS families [27]. They observed that, unlike the Mendelian distribution of grandparental alleles seen in control families, LQTS alleles were more frequently of maternal than paternal origin. Additionally, the excess of disease-causing alleles of maternal origin was most pronounced in LQT1 [27]. The mechanism resulting in the higher transmission rate of maternal LQTS alleles, highly significant in LQT1 families, is still unclear. Although abnormal imprint resetting could lead to early death of embryos [28], the specific loss of male vs. female embryos that could explain the disproportion seem difficult to conceive [27]. They also considered that gender differences described in LQTS expression are insufficient to support the theory of a reduction in the number of male carriers of reproductive age, compared with female carriers [27]. In contrast, they favor the hypothesis that the dysfunction of potassium channels could have a major role in preferential maternal transmission in LQTS [27,29,30,31]. In our cohort, all index cases but one were women. Nonetheless, when analyzing families, the same proportion of gender representation among carriers was found (50% each). 

Apart from than, although young men have traditionally been considered to be at higher risk of LAEs, at least at young age [32], our findings make us wonder if this could be actually be extrapolated to the *KCNQ1* G168R variant carriers. An interesting study in a large population of 1051 genetically-confirmed patients with LQT1 found, supporting prior observations, a significantly higher rate of LAEs in men, especially prior to puberty [33]. They hypothesize that may be partially explained by differences in the level of physical activity between genders during childhood. After the onset of adolescence, an increase in the levels of testosterone, which was shown to shorten action potential duration and ventricular repolarization [34,35] may result in a reduction in the risk for arrhythmic events in men. Imprinting mechanisms previously described may also play an important role. In addition, the risk for LAEs was increased among women only with variants in C-loop domains (S2–S3 and S4–S5), unlike men with high risk even among those previously considered as lower-risk variants [33]. However, in our cohort, clinical phenotype seems to be more severe in women, as all the registered SCD occurred in woman. Moreover, penetrance in women was a 100%, whereas in men it was below 40%.

## 5. Limitations

Family screening was not available for all relatives. Information about genotype and phenotype of certain relatives was impossible to obtain (due to geographic limitations or patients’ refusal). Description of larger families would support and help to establish further genotype-phenotype strong correlations. Genotype-phenotype associations observed in this manuscript are not applicable to other variants in either this gen or other LQTS related genes. Many unexplained SD occurred without clinical or genetic confirmation of LQST and this could implicate survival bias. Moreover, other factors that could modify the phenotype expression, such as socio-economic status, environment or lifestyle, have not been analyzed. 

## 6. Conclusions

*KCNQ1* G168R is LQTS1 a pathogenic variant with a high penetrance among women and mild penetrance among men. Unlike other pathogenic variants described in membrane spanning domain of *KCNQ1* gen, the risk for LAEs in *KCNQ1* G168R carriers should not be assumed to be low and risk stratification should always be carefully preformed.

## Figures and Tables

**Table 1:** Index patients and family characteristics of *KCNQ1* G168R carriers.

Index Patient	Gender	Warning Symptoms	Aborted Sudden Deaths in the Family (Gender)	Sudden Deaths in the Family (Gender)	Male Carriers with LQTS Phenotype	Female Carriers with LQTS Phenotype
1	Female	Syncope	1 (Female)	3 (Female)	100% (1/1)	100% (1/1)
2	Female	Aborted SD	1 (Female)	3 (Female)	None	100% (3/3)
3	Female	Aborted SD	2 (Female)	0	12.5% (1/8)	100% (5/5) *
4	Male	None	0	3 (Female)	50% (3/6) *	100% (2/2)
5	Female	None	0	0	100% (1/1)	100% (3/3)
6	Female	None	0	0	None	100% (1/1)
7	Female	None	0	0	None	100% (1/1)

* Two obligate carriers (one women from family 3 and one male from family 4) died without clinical evaluation or available EKGs (unknown phenotype).
